# Synthesis and Performance of Large-Scale Cost-Effective Environment-Friendly Nanostructured Thermoelectric Materials

**DOI:** 10.3390/nano11051091

**Published:** 2021-04-23

**Authors:** Farheen F. Jaldurgam, Zubair Ahmad, Farid Touati

**Affiliations:** 1 Department of Electrical Engineering, College of Engineering, Qatar University, Doha 2713, Qatar; fj1912900@student.qu.edu.qa (F.F.J.); touatif@qu.edu.qa (F.T.); 2 Qatar University Young Scientist Center (YSC), Qatar University, Doha 2713, Qatar; 3 Center for Advanced Materials (CAM), Qatar University, Doha 2713, Qatar

**Keywords:** thermoelectric materials, nanostructures, large-scale production techniques, ecofriendly, powder metallurgy, printable techniques

## Abstract

Thermoelectricity is a promising technology that directly converts heat energy into electricity and finds its use in enormous applications. This technology can be used for waste heat recovery from automobile exhausts and industrial sectors and convert the heat from solar energy, especially in hot and humid areas such as Qatar. The large-scale, cost-effective commercialization of thermoelectric generators requires the processing and fabrication of nanostructured materials with quick, easy, and inexpensive techniques. Moreover, the methods should be replicable and reproducible, along with stability in terms of electrical, thermal, and mechanical properties of the TE material. This report summarizes and compares the up-to-date technologies available for batch production of the earth-abundant and ecofriendly materials along with some notorious works in this domain. We have also evaluated and assessed the pros and cons of each technique and its effect on the properties of the materials. The simplicity, time, and cost of each synthesis technique have also been discussed and compared with the conventional methods.

## 1. Introduction

Thermoelectric devices are compact solid-state devices that are low maintenance and have low levels of noise and vibrations along with precise control over any slight temperature changes. However, current low thermal and electrical efficiencies are due to uneven temperature distributions. Developing high-performance thermoelectric materials has a lot of potential future. Their current lower conversion efficiencies have limited them to niche applications. Over the past decades, several efforts have been made to improve thermoelectrics efficiency, mostly based on nanostructuring, molecular rattling, doping, and so on. These methods have shown excellent results on the laboratory scale. Scaling these methods is hindered by manufacturing challenges, power conversion efficiency, complexity, longer processing times, and system and material costs. The efficiencies of thermoelectric materials are measured in terms of well-defined dimensionless factor ZT, which is known as the figure of merit [1] and given by:

(1) $$\mathrm{ZT}=\frac{S^{2}}{\rho \kappa }T$$

where S is the Seebeck coefficient, ρ is the electrical resistivity, κ is the thermal conductivity, and T is the absolute temperature. Few thermoelectric materials such as PbTe and SiGe have shown figure of merit (ZT) values of around 2 [2,3]. Existing commercial-based thermoelectric generators (TEGs) are mostly bismuth-based and are predominantly used for localized heating, cooling, and deep space applications. Many other thermoelectric materials are being explored for power generation applications, such as GeTe [4], PbTe [5], and silicides [6]. The high toxicity and scarcity of raw materials such as Ge, Te, Pb, etc., used to compose the thermoelectric material make the processing expensive and is an obstacle for the widespread commercialization of thermoelectric generators or devices.

Large-scale synthesis and fabrication techniques for thermoelectrics are essential for successfully integrating TEGs in wide applications. There are different kinds of TE synthesis and fabrication techniques, from chemical-based methods to metallurgy-based methods and printable techniques. However, most of the current practices available have one or more disadvantages in terms of cost, reliability, time, efficiency, controllability, complexity, or high energy consumption. Researchers have explored this domain, and various novel successful synthesis and fabrication techniques have been proposed to tackle the obstacles that have restricted thermoelectrics. The first goal for large-scale commercialization is to devise a material that is easily assessable and low-toxic, and the second is to find an appropriate synthesis technique that is simple, fast, inexpensive, reliable, and reproducible. The fabrication methods suitable for large-scale production need to be explored thoroughly in all the concerned aspects. In our previous paper [7], we have dealt with identifying low-toxic and earth-abundant nanostructured materials in different operating temperatures. Yet, these materials are behind conventional materials and require further enhancement for successful embedment.

The best thermoelectric material should exhibit photon-glass/electron-crystal behavior (PGEC) [8]. That indicates that the material should have the electrical properties of a crystal and thermal properties of glass. The basic concept of this PGEC is to assert that the semiconductors, while largely blocking the heat transfer by the lattice (similar to the glass), are very efficient at transmitting charge carriers as in a crystal. However, it is challenging to design a material that has very low heat transport while maintaining good electrical conductivities. The discovery of nanostructured thermoelectric materials such as nanowires, quantum dots, superlattices, and nanocomposites has remarkably progressed the power conversion efficiencies of the thermoelectric materials. Nanostructuration aids in achieving the very specific group of properties essential for high-performance TE. Nanostructuring helps increase the figure of merit (ZT) of the thermoelectric material by drastically lowering the lattice thermal conductivities.

The traditional methods used for the fabrication or synthesis of thermoelectric materials are one or more combinations of the following: hot-pressing [9], mechanical alloying [10], microwave sintering [11], simple chemical route synthesis [12], ball milling [13], solid-state synthesis [14], etc. The shortcomings of these methods such as limited reproducibility, higher costs, and longer processing times have limited them to be applicable in large-scale production. In our report, firstly, we discuss the recent advancements in different large-scale synthesis and fabrication for nanostructured environment-friendly TE materials in terms of the current efficiency achieved by each method given by the figure of merit. Then, we explain in detail the various works conducted in each TE batch synthesis and fabrication process. Finally, we summarize and compare the benefits and limitations of each technique and provide a direction toward future developments. It is important to mention that the review of the materials was done based on the factor that includes performance, large-scale synthesis, cost-effectiveness, and relatively low toxicity. Of course, the materials’ toxicity is sometimes tricky to avoid if we consider all four factors altogether.

## 2. Recent Advancements in Large-Scale Thermoelectric Synthesis and Fabrication Methods

Table 1 gives a detailed summary of the processing cost, time, scalability, the figure of merit ranges, strengths, and drawbacks of different techniques. The microwave (MW)-assisted hydro or solvothermal method is mostly used to synthesize nanostructures in inorganic materials such as Bi_2_S_3_, SnTe, etc. Solvothermal or hydrothermal is an old method that has proven advantageous in producing highly crystalline nanostructures with high purity, narrow size distribution, and low aggregation. This MW-hydrothermal/solvothermal method combines and possesses the merits of both the hydrothermal/solvothermal and microwave approaches. These methods combined with microwave synthesis provide short sintering times, low temperatures, economic processing, rapid diffusion energies, and the existence of highly crystalline nanostructures. These are the features that are essential for successful large-scale manufacturing. Existing works show that the figure of merit for the materials with microwave-assisted synthesis is close to 2 (ZT ≈ 2 at 873 K for SnSe [15]) with maximum scalability of up to 10 g. However, this method requires a long reaction time, complex equipment, and high energy consumption. Some of the other materials that can be synthesized from these methods are metal oxides, hydroxides, metal composite oxides, inorganic biomaterials, and metal sulfides and can be used in areas such as gas sensors, photocatalytic, electrochemical, solar cell, and optical applications [16].

Chemical synthesis involves solution-phase chemical reactions using precursors at appropriate experimental conditions. Each chemical synthesis route differs from the other, indicating diverse synthesis environments and chemicals involved in each method. These methods are mainly used to prepare the 1D and 2D nanostructures such as nanoflakes, nanotubes, nanorods, nanoparticles, etc., that are difficult to be structured by top–down approaches. These methods have superior controllability with high reproducibility. Various chemical synthesis methods have also been examined in thermoelectrics and have proved successful in synthesizing nanostructures in inorganic TE materials such as Bi_2_Te_3_, chalcogenides, etc. The current figure of merit of these methods is in the range 1–1.2 (ZT = 1.18 at 500 K for Bi_2_Te_2.5_Se_0.5_ [17]) and provides better mechanical properties with good characterization and scalability up to 20 g. However, these chemical methods are moderately developed, and the synthesis of heterostructures is still not achieved. The development reached now has come a long way in enhancing the TE performance. The thermal conductivities and Seebeck coefficient have been notably optimized, whereas the electrical conductivities are still on the lower side due to the influence of grain boundaries and insufficient contact. Enhancing electrical conductivities to build high-quality bulk samples or films with various nanostructures and implementing them on a large scale is the most challenging step for these chemical methods. The advance of robust synthetic routes, optimized bulk sample preparation, precise micro-processing techniques, etc., would provide the attention these chemical methods deserve in the TE industry for wide-ranging applications.

The powder metallurgy process involves mixing alloy or elemental powders, compaction, sintering, and finishing. Several powder metallurgical processes such as gas atomization and cryogenic grinding provide good scalability with efficient thermoelectric properties. These methods produce high-quality powders with high scalability (up to 3–5 kg/min). Gas atomization methods have superiority in terms of scalability, whereas cryogenic grinding is better in terms of thermoelectric performance (1.55 at 825 K for Yb single-filled CoSb_3_ [18]). These methods have efficient material utilization, design of complex shapes, good surface finishing, energy-efficient, environmentally friendly, and most suitable for producing quantities in large quantities. These are not well-known methods due to limitations in variability and the high initial cost of powder, even with these exceptional benefits. These methods are extensively used in the automobile industry.

**Table 1:** Summary of recent progress in prominent large-scale synthesis methods.

Technology	Materials	Processing Cost	Time	Scalability (Max Weight Per Batch)	Ztmax (Material-Ref)	Strengths	Drawbacks
Microwave-assisted hydrothermal/solvothermal	Inorganic (Bi_2_S_3,_ SnTe, α- MgAgSb,etc) nanostructures	Medium	Moderate	≈10 g	2.2 (SnSe [15])	Lower operating temperatures, minimum material loss, good dispersion, and eco-friendliness, highly crystalline nanostructures	Inability to observe and monitor the reaction process
Chemical synthesis	Inorganic (Bi_2_Te_3_, Copper-based)	Low	Moderate	≈20 g	1.2 (Bi_2_Te_2.5_Se_0.5_ [17])	Simple, inexpensive, better mechanical properties, better characterization	Controlling the parameters of deposition is difficult to achieve
Gas Atomization	Inorganic	Low	Ultra-fast	3–5 Kg/min	≈1 (Bi_2_Te_3_+ 75% Sb_2_Te_3_ [19])	High-quality pure powders, high powder flow rates, economical, very high scalability	Powder properties vary with the equipment from different suppliers
Cryogenic grinding	Inorganic, hybrid	High	Fast	High	1.5 (Yb single-filled CoSb_3_ [18])	Fine sintered powders, improved mechanical abilities, power saver, no oxidation	Formation of ice around the delivery nozzle and piping system blocks delivery of liquid nitrogen

Conventional TEG fabrication methods involve electrically connecting the diced pellets with metal electrodes fashioned as a sandwich in two ceramic plates. These methods involve expensive equipment, high temperatures, and are not particularly suitable for flexible electronics applications. Several printing fabrication methods, such as inkjet printing, screen printing, dispenser printing, and photonic sintering, are utilized primarily for wearable and portable electronic applications. These methods are used for organic, inorganic, and hybrid organic–inorganic TE materials. They are simple, easy, quick, durable, and do not require complex post-processing. Printable methods also have the advantage of low-cost equipment, low-temperature, low-material wastage, vacuum-less fabrication, and high reproducibility. Compared to other techniques available for large-scale synthesis, these methods are still in their infancy and have low conversion efficiencies and figure of merit values (between 0 and 1). Some challenges faced in these methods are non-uniform deposition, low-resolution, and rough surface of the substrates. Even with the fast and extensive research, there is still a long way ahead for these methods before being employed in large-scale practical environments. Detailed evolution of the different large-scale, cost-effective, environment-friendly synthesis and fabrication methods are discussed in the next section. Table 2 gives a summary of the current progress made in different large-scale fabrication techniques.

## 3. Various Synthesis and Fabrication Techniques for Large-Scale Production

### 3.1. Microwave-Assisted Solvothermal/Hydrothermal Synthesis

**Figure 1:**
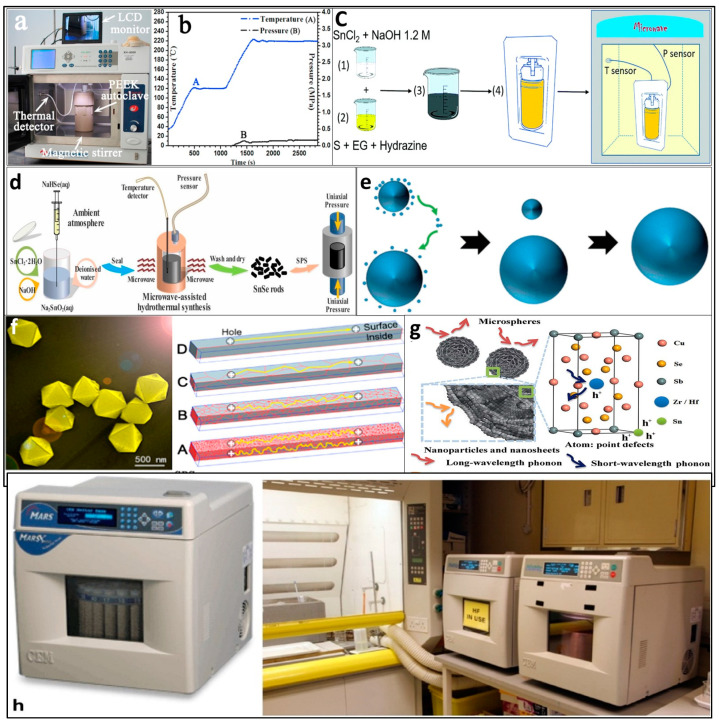
(**a**) Picture depicting main units of the microwave-assisted hydrothermal (MH) synthesis system. Reproduced with permission from [36]. Copyright Elsevier, 2016, (**b**) The temperature and pressure curve as a function of the reaction time. Reproduced with permission from [36]. Copyright Elsevier, 2016, (**c**) Microwave-assisted hydrothermal synthesis of Sn1-δS powder. Reproduced with permission from [41]. Copyright 2020, Royal Society of Chemistry, (**d**) Schematic diagram of fabrication of Tin selenide (SnSe) pellets by microwave-assisted hydrothermal synthesis and SPS (spark plasma sintering). Reproduced with permission from [42]. Copyright Royal Society of Chemistry, 2020, (**e**) The classical Ostwald ripening progress in CuFeO2 powders synthesized by MH reaction. Reproduced with permission from [39]. Copyright Journal of the Ceramic Society of Japan, 2019, (**f**) Tin telluride nanoparticles (NP) synthesized by MH method and building blocks of SnTe after SPS. Reproduced with permission from [36]. Copyright Elsevier, 2016, (**g**) Point defects in Zr, Sn or Hf, Sn co-doped Cu3SbSe4 nanoparticles. Reproduced with permission from [38]. Copyright Elsevier, 2020, (**h**) Typical MARS equipment for microwave hydro/solvothermal processing (cost around 30,000 USD. Reproduced with permission from [43]. Copyright The Development and Application of Microwave Heating, 2012.

Researchers have suggested various novel methods for the large-scale synthesis of low-toxic materials. Improving the thermoelectric properties requires lowering the thermal conductivities by nanostructuring of the materials. Initial works focused on the large-scale synthesis of 1D structures such as nanowires and nanotubes. Solvothermal or hydrothermal synthesis is one route that is effective in the batch production of thermoelectric materials. Solvothermal synthesis is a chemical reaction in a solvent at above boiling point temperatures and 1 bar pressures. The main difference between the hydrothermal and solvothermal processes is that the solvent used in the hydrothermal method is water. The hydrothermal and solvothermal methods are available for the past three decades to synthesize nanostructured materials such as β-In_2_S_3_ [23], Bi_2_S_3_ [24,25,26], Sb_2_S_3_ [27], Bi_2_Se_3_ [28,29], and Sb_2_Te_3_ [30]. Figure 1a depicts the main units of the microwave-assisted hydrothermal (MH) synthesis system and Figure 1b illustrates the temperature and pressure curve as a function of the reaction time. Figure 1f demonstrates the tin telluride nanoparticles synthesized by the MH method and building blocks of SnTe after SPS. Zhu et al. [31] described a simple hydrothermal process for synthesizing ultralong Sb_2_S_3_ of about 200 nm diameter and 100 µm length. In this method, ethylene glycol was used for assistance, and the resultant material was synthesized from samples SbCl_3_ and Na_2_S of weights 0.346 g and 1.103 g, respectively, in the presence of distilled water. Another work also utilized a synthetic hydrothermal method for preparing 10 g of Cu_2_ZnSn(S, Se)_4_ nanocrystals per batch to reach a ZT of ≈0.5 [32]. Two-dimensional (2D) nanoplates of Ge-doped tin selenide (Sn_0.97_Ge_0.03_Se) prepared from simple hydrothermal synthesis followed by SPS demonstrated a high figure of merit of ≈2.1 at 873 K [15]. Ultralong Bi_2_S_3_ nanoribbons of thickness 20–80 nm and widths of 50–300 nm were synthesized using a simple solvothermal process with a solvent of glycerol and aqueous NaOH solution mixture [33]. Figure 1c illustrates the microwave-assisted hydrothermal synthesis of Sn_1-__δ_S powder.

The microwave irradiation method is a simple, fast, and economic process that uses high-frequency waves to heat the materials in a short time at high temperatures. This method was basically used for synthesizing graphene-based materials. Still, lately, this has been used to synthesize nanostructures, since it provides efficient uniform internal in-core volumetric heating by direct coupling of microwave energy to the elements in the reaction medium. This uniform fast, homogeneous heating minimizes any thermal gradients and provides consistent growth conditions, nucleation, and highly crystalline materials. Microwave irradiation methods were used to prepare crystalline Bi_2_S_3_ nanorods from thiourea and bismuth citrate [34]. α-MAS or α- MgAgSb is a promising earth-abundant and low-toxic p-type thermoelectric material. The large-scale synthesis is challenging, firstly due to the extended period for the preparation of α-phase by conventional methods and secondly due to poor electrical properties from the severe bipolar effect. Xin et al. [35] prepared α-MAS with rapid microwave synthesis and then powdered by mortar–pestle. Tin telluride (SnTe) nanocrystals were synthesized by a facile solvothermal method and then mixed with the α-MAS powder via ball milling in high-purity Argon gas. The resultant α-MAS/SnTe composited system had a figure of merit close to unity (≈1) with larger Seebeck coefficients than pristine α-MAS.

Hydrothermal synthesis has advantages as lower operating temperatures (<300 °C—beneficial to materials that are not stable over elevated temperatures), minimum material loss, good dispersion, and eco-friendliness. The only problem with this method is the need for expensive equipment and the inability to observe and monitor the reaction process. Combining microwave with hydrothermal would result in short sintering times, low temperatures, economic processing, rapid diffusion energies, and the existence of highly crystalline nanostructures. Tine selenide (SnTe) synthesized using a facile, ultra-fast, and simple microwave hydrothermal method enhanced the photon scattering effect and resulted in a maximum ZT value of 0.49 at 803 K with a low thermal conductivity of 0.60 W m^−1^ K^−1^ [36]. The addition of Se and Cd as dopants introduced strong point defect scattering in the Sn_0.98_Cd_0.02_Te_0.9_Se_0.1_ sample synthesized by the microwave-stimulated solvothermal method and spark plasma sintering with a maximum ZT of 0.78 at 773 K [37]. In a similar work, the Sn and Zr or Hf co-doped p-type Cu_3_SbSe_4_ were synthesized in a 500 mL special Teflon autoclave, placed in a microwave oven [38]. Point defects in Zr, Sn, or Hf, Sn co-doped Cu_3_SbSe_4_ nanoparticles can be seen in Figure 1g. Then, the powders were treated in Argon flow at 573 K for 2 h and then spark plasma sintered under 40 MPa axial pressure at 623 K for 5 min to give the highest ZT of 0.82. Zhai et al. [39] found that the grain growth in CuFeO2 powders synthesized by the microwave hydrothermal (MH) reaction satisfied the theory of classical Ostwald ripening, and a pure rhombohedral phase in the material has been formed, as shown in Figure 1e. Nanostructured particles of size in the range 50–100 nm were formed in Ga-doped ZnO made by the MH technique [40]. A one-step MH procedure was used to prepare phase-pure polycrystalline Sn_1-__δ_S with a high ZT of 0.76 at 523 K due to relatively good mobility and high carrier concentration [41]. Phase-pure tin selenide (SnSe) micro rods with a high maximum ZT of 1.08 were prepared by a combination of microwave-assisted hydrothermal synthesis and SPS (spark plasma sintering), as shown in Figure 1d [42]. Figure 1h shows a typical MARS (CEM Corp.) microwave-assisted hydrothermal (MH) synthesis systemfor microwave hydro/solvothermal processing Thus, microwave-assisted solvothermal or hydrothermal methods are promising techniques to synthesize efficient and high-performance thermoelectric materials on a large scale.

### 3.2. Melt-Spinning

Melt-spinning (MS) is a common and economical spinning method to fabricate polymer nanofibers [44]. This technique used for the rapid solidification of liquids and the high rate of cooling in the melt-spinning process assists in forming refined nanostructures and better amorphous phases in TE materials on a large scale. Tang et al. used this melt-spinning method followed by spark plasma sintering (SPS) to synthesize several Earth-abundant high efficient nanostructured TE materials such as higher manganese silicides (ZT = 0.62 at 800 K [45]), bismuth telluride alloys (ZT = 1.5 at 390 K [46]), beta-Zn_4_Sb_3_ (ZT = 1.3 at 700 K [47]), and n-type CoSb_3-_based skutterudites (ZT = 1.3 at 800 K [48]). Optimization in cooling rates during the melt-spinning process further increased the ZT ≈0.83 at 800 K in p-type higher manganese silicides (HMS) [49]. We used MS combined with hot pressing to produce high efficient Bi and In co-doped tin telluride (SnTe) nanomaterial with a high ZT of 1.26 at 900 K [50]. MS followed by SPS reduced the thermal conductivity to 0.57 Wm^−1^ K^−1^ and ZT ~ 0.95 at 800 K in the case of Sb-doped SnTe based nanomaterials [51]. This melt-spinning method is an efficient route to develop high-performance nanostructured TE materials on a large scale.

### 3.3. Chemical Synthesis

#### 3.3.1. Colloidal Synthesis

**Figure 2:**
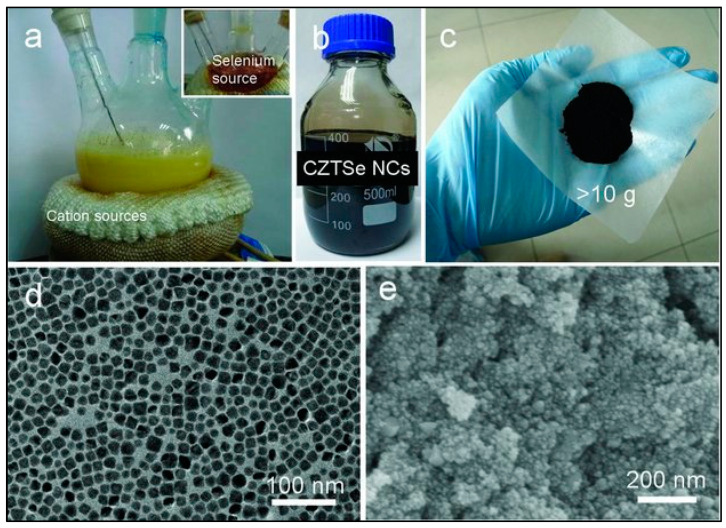
(**a**) Large-scale colloidal synthesis of copper zinc tin selenide (CZTSe) (pictures of the reaction flasks), (**b**) The synthesized non-stoichiometric CZTSe nanocrystals dissolved in hexane, (**c**) More than 10 g synthesized surface-clean CZTS nanoparticles, (**d**,**e**) Transmission Electron Microscopy (TEM) and Scanning Electron Microscopy (SEM) images of the synthesized nanoparticles. Reproduced with permission from [52]. Copyright Wiley Online Library, 2017.

Some works also suggest the colloidal synthesis method for large-scale synthesis. Colloidal synthesis is a conventional method to synthesize uniform-sized inorganic nanocrystals in a size-controlled fashion. It is a well-established and straightforward wet chemistry process. The colloidal method also provides better characterization of size-dependent properties, which is challenging using top–down physical approaches. First, 10 g weight of non-stoichiometric copper zinc tin selenide (CZTSe) nanocrystals were prepared by the colloidal synthesis in work [52]. Figure 2 shows the complete process of the large-scale colloidal synthesis of CZTSe. The CZTSe materials showed suppressed thermal conductivities due to the increased crystalline boundaries. Another work used a large-scale, inexpensive, and simple colloidal synthetic route to form uniform-sized Bismuth nanocrystals of size range 6–27 nm by reducing the bismuth thiolate with tri-n-octyl phosphine [53]. The bismuth nanocrystals demonstrated very high electrical conductivities (10^4^–10^5^ S-m^−1^) and very low thermal conductivities (0.35 W m^−1^ K^−1^) [53]. N-type nanostructured ultrathin Bi_2_Te_3_ nanoplates synthesized on a large scale using colloidal synthesis, and SPS showed a high ZT value of 0.62 in the sample sintered at 250 °C [54]. Similarly, 10 g of cobalt-doped Cu_2_SnSe_3_ nanocrystals were synthesized per one run by using this colloidal method followed by spark plasma sintering to reach a high ZT value of 0.63 at 715 K [55]. Some other works have also used this colloidal method to synthesize Cu_2_SnSe_3_ and enhance its thermoelectric properties [52,56,57]. Yang et al. used tellurium oxide as a tellurium precursor to synthesize monodispersed nanocrystals of another similar material Cu_2_SnTe_3_ [58]. Yin et al. [59] synthesized different nanostructures such as Cu_1.29_Te nanocubes, nanosheets, and nanorods; Cu_1.43_Te hexagonal nanoplates; SnTe nanorods; and Cu_2_SnTe_3_ nanocubes by carefully controlling the amount of tin precursor and reaction temperature in a colloidal synthesis method. B. Xu et al. reported low-temperature and scalable (ca. 11.0 g per batch) colloidal processing and then used a spark plasma sintering route to fabricate highly porous Bi_2_Te_2.5_Se_0.5_ nanocomposite thermoelectric materials to yield a maximum ZT of 1.18 in the intermediate temperature range [17]. This is the highest value of ZT reported in the case of large-scale fabrication of low-toxic and abundant thermoelectric materials. Additionally, better mechanical properties are observed in the nanostructures of thermoelectric materials synthesized by colloidal synthesis compared to the solid-state reaction method [60].

#### 3.3.2. Wet Chemical Method

**Figure 3:**
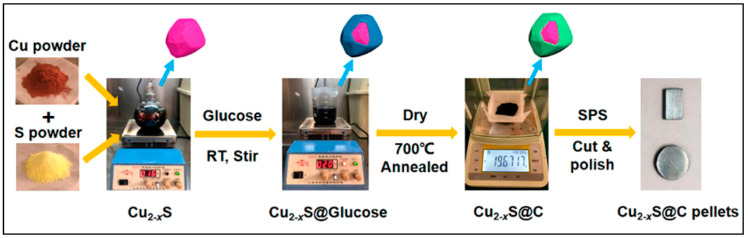
Schematic illustration of the carbon-encapsulated copper sulfide (Cu_2−x_S@C) composite synthesis process. Reproduced with permission from [61]. Copyright ACS Publications, 2019.

Wet chemical synthesis is used to synthesize nanostructured materials using liquid-phase precursors in the solution phase under certain proper and pre-defined experimental conditions. Carbon-encapsulated copper sulfide chalcogenide (Cu_2-x_S) was synthesized using a scalable wet chemical method to result in enhanced thermoelectric properties (ZT ≈ 1.04 at 773 K and thermal conductivity ≈ 0.22 Wm^−1^ K^−1^) [61]. In this method, copper and sulfur powders were chemically processed to form Cu_2-x_S and dispersed into glucose in ethanol solution using ultrasonic agitation. Later, it was dried and annealed at 700 °C for 2 h to attain Cu_2−x_S@C. Lastly, this powder was then spark plasma sintered into a pellet by loading into a graphite die of diameter 12 mm at temperature 420 °C under 70 MPa for 5 min. Figure 3 illustrates the complete synthesis process of Cu_2-x_S using the wet chemical method. Even though the chemical methods are low-cost and fast, their only disadvantage is their repeatability and reproducibility of thermoelectric properties after sintering, making it hard for its practical implementation in large-scale thermoelectric fabrication industries. With many more advancements and developments, chemical methods would prove an imperative method for practical, scalable fabrications without compromising the thermoelectric performance.

#### 3.3.3. Solution Synthesis

**Figure 4:**
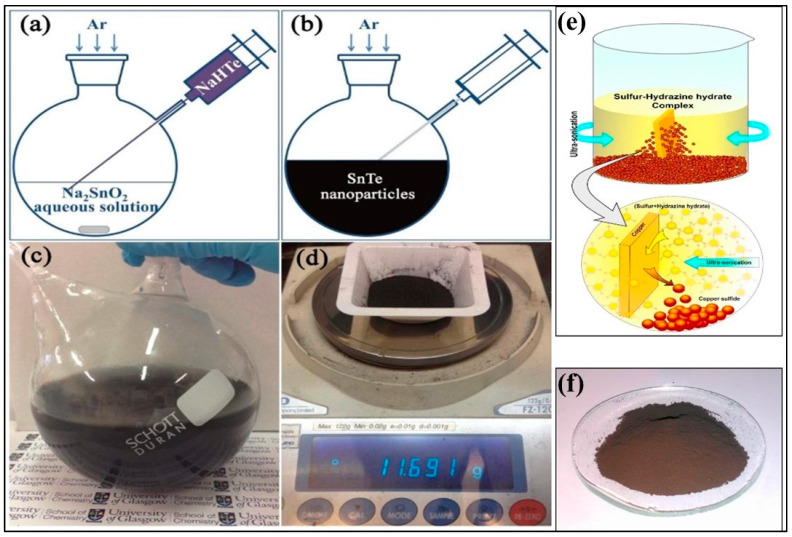
Solution synthesis of tin telluride (SnTe) nanoparticles, (**a**) Na_2_SnO_2_ aqueous solution is injected with NaHTe solution, (**b**) Formation of tin telluride (SnTe) nanoparticles, (**c**) Photograph showing the solution after a scale-up synthesis, and (**d**) Typical yield of tin telluride (SnTe) nanoparticles prepared in a one-pot synthesis. Reproduced with permission from [62]. Copyright MDPI, 2017, (**e**) Bulk synthesis of copper sulfide by the ultrasonic experimental setup. (**f**) Photograph of prepared Copper sulfide using 1 M sulfur solution. Reproduced with permission from [63]. Copyright Elsevier, 2017.

The decomposition of molecular or atomic monomers in liquid-phase methods occurs within a solvent when heated to specific temperatures. The selection of suitable concentrations and types of chemicals used, including precursors, solvents, redox agents, and surfactants, is essential to obtain high crystalline nanoparticles (NP). The temperature profile, the temperature of additives to the reaction mixture, and consideration time also influence the quality of the nanostructures formed. The method should be simple, with chemicals ideally accessible commercially, stable at room temperatures, safe, low-cost, eco-friendly, and insensitive to air, light, and moisture. The purity of compounds is essential to ensure the repeatability and reliability of the final product, since the impurities play a vital role during the NP synthesis and influence crucial thermoelectric properties such as electronic band structure and charge carrier concentration of the composed TE material. An exhaustive collection of solution-based procedures is available to produce NP for diverse, high competent thermoelectric materials such as chalcogenides and novel metals. The control and growth of nanostructures in chalcogenides are relatively favorable due to their ease of growth at low temperatures, the availability of a broad range of commercial precursors, and their numerous thermoelectrics applications. Tellurides and selenides are preferred for their large conductivities, low band gaps, and thermal conductivities. A quick, simple, and inexpensive one-pot solution route was used to straightforward synthesize tin telluride nanoparticles in gram quantities (≈12 g per run for 2 h growth), as shown in Figure 4a–d [62]. Then, the SnTe nanoparticles (NP) were spark plasma sintered to form dense nanostructured SnTe pellets. Mulla et al. [63] demonstrated a simple chemical method to fabricate copper sulfide on a large scale with a very high yield. Figure 4e–f demonstrates the bulk synthesis of copper sulfide by the ultrasonic experimental setup. These methods effectively synthesize similar chalcogenide TE nanomaterials. n-type chlorine-containing tin selenide (SnSe) NPs were synthesized 10 g per batch using an aqueous solution and hot pressing [64]. Bismuth(III) 2-ethyl hexanoate was used as a cationic dopant precursor to solution synthesize n-type Bi-doped SnSe to reach a Seebeck coefficient of ≈900 μV K^−1^ at room temperature [65]. Another work used a controlled single-step solution method to synthesize SnSe/eGO (reduced graphene oxide) nanocomposites to yield a very low lattice thermal conductivity of 0.36 Wm^−1^ K^−1^ and maximum ZT of 0.91 at 823 K [66]. This solution processing the TE materials in nanostructured forms enables the effective use of printing technologies for superior throughput fabrication of cost-effective and flexible thermoelectric devices.

### 3.4. Powder Metallurgy

#### 3.4.1. Gas Atomization

**Figure 5:**
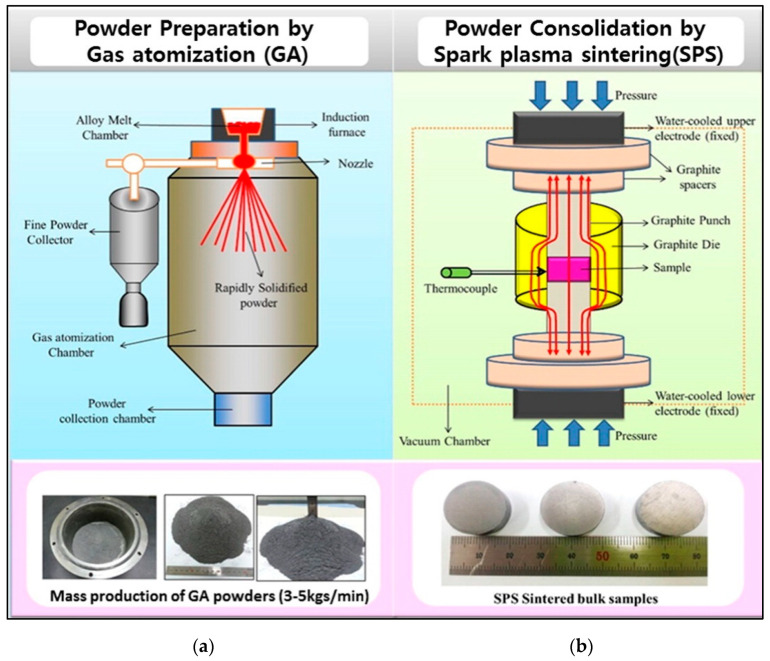
(**a**) Large-scale synthesis of Bi-Sb-Te alloys powder (bismuth antimony telluride) by gas atomization (GA) (3–5 kg/min) (synthesized Bi_2_Te_3_ + 75%Sb_2_Te_3_ powder can also be seen). (**b**) Spark plasma sintering (SPS) is used to consolidate the resultant nanopowder (the spark plasma sintered bulk samples can also be seen). Reproduced with permission from [19]. Copyright Elsevier, 2016.

Gas atomization (GA) is a process to synthesize high-quality powders. In this process, the molten materials are atomized with inert gas jets into fine material droplets that cool down in the atomized tower during their fall. This powder is collected in a capsule and then compacted using various techniques such as ball milling, spark plasma sintering, etc. Hong et al. [67] prepared n-type SbI_3_-doped 95%Bi_2_Te_3_ –5%Bi_2_Se_3_ by GA and extrusion at 450 °C in the ratio 25:1 to form bars of length ranging from 70 to 780 mm. Due to the dynamic recrystallization during hot extrusion, the microstructure of the bar indicated fine and homogeneous distribution along its length. From the extension of this work, the optimum dopant concentration was found to be 0.04 wt % SbI_3_ [68]. In another work, p-type Te-doped Bi_2_Te_3_–Sb_2_Te_3_ were prepared using GA, which was followed by a spark plasma sintering process [69]. This work found a reduction in hardness with an increase in sintering temperatures. Gas atomization combined with ball milling and then followed by SPS was used to synthesize high-performance large-scale production (3–5 kg/min) of p-type Bi_2_Te_3_ + 75%Sb_2_Te_3_ with the figure of merit close to unity at 350 K [19]. Figure 5a is the schematic illustration of gas atomization synthesis of p-type Bi_2_Te_3_+75%Sb_2_Te_3_. Figure 5b shows the consolidation of the powder into pellets by using spark plasma sintering. Compared to other conventional large-scale production techniques, gas atomization can fabricate larger quantities of materials in short periods (3–5 kg/min [19]).

#### 3.4.2. Cryogenic Grinding

**Figure 6:**
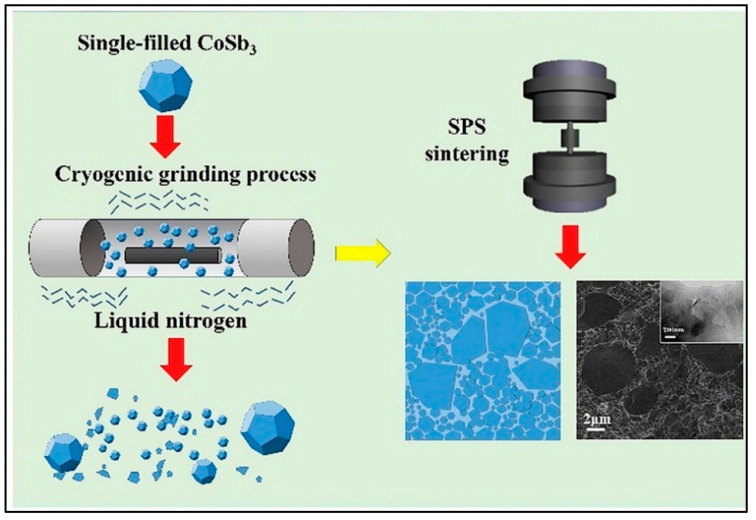
Cryogenic grinding of Yb single-filled CoSb3 into finer particle sizes in the presence of liquid nitrogen. The spark plasma sintering of the powder can also be seen. The SEM image of the material at 2 µm is also included. Reproduced with permission from [18]. Copyright Elsevier, 2019.

Cryogenic grinding (CG) or freezer milling is the process of chilling or cooling material and then reducing it into finer particle sizes. It is done in the presence of liquid nitrogen, liquid carbon dioxide, or dry ice. This method was first used in thermoelectrics to synthesize Bi_2_Te_3_ nano-sized powders in the presence of liquid nitrogen [70]. No amorphous state or phase transformation was observed during CG and compared with spark plasma sintering and high-energy ball milling, and this method produced better sinterability and finer powders. Hybrid organic composites prepared by CG showed enhanced carrier mobility and carrier concentration due to the orderly and homogeneous dispersion [71,72]. Skutterudites prepared using CG-SPS demonstrated better figure of merit values (1.42 at 850 K for β-SiC/Yb_0.3_Co_4_Sb_12_ nanocomposites [73] and 1.55 at 825 K for Yb single-filled CoSb_3_ [18]) due to enhancement in the Seebeck coefficient and lowering of lattice thermal conductivities by the energy-filtering effect. Figure 6 shows the schematic illustration of the preparation of Yb single-filled CoSb_3_ via cryogenic grinding and spark plasma sintering. This method is reproducible, and it also improved the mechanical properties such as flexural strength, fracture toughness, and Vickers hardness of the thermoelectric material [74].

### 3.5. Printable Technologies

Printing technology is a new technique for sustainable fabrication that has enabled a wide range of conformable and biocompatible systems. Printing helps vacuum less, low-temperature, and low-cost custom-made thermoelectric devices with better flexibility, low material waste, and lightweight devices. These methods avoid heavy and expensive machinery and higher processing temperatures, reducing cost and times of production. Printing methods have lately been used to manufacture solar cells, thin-film transistors, memristors, light emitting diodes (LEDs), and many more electronics and electrical components. Various printing techniques are inkjet printing, screen printing, dispenser printing, stereolithography, brush printing, and roll-to-roll printing. Most of the printing techniques are used to print organic materials on flexible bio-compatible substrates. Organic compounds are flexible, solution-processable, abundant, and environmentally benign but have lower power conversion efficiencies. To fill this void, organic–inorganic hybrid composites are employed that have better power conversion efficiencies and ease of processing. Our report summarized inkjet printing, screen printing, and dispenser technologies due to their prominence and broader applications.

#### 3.5.1. Inkjet Printing

**Figure 7:**
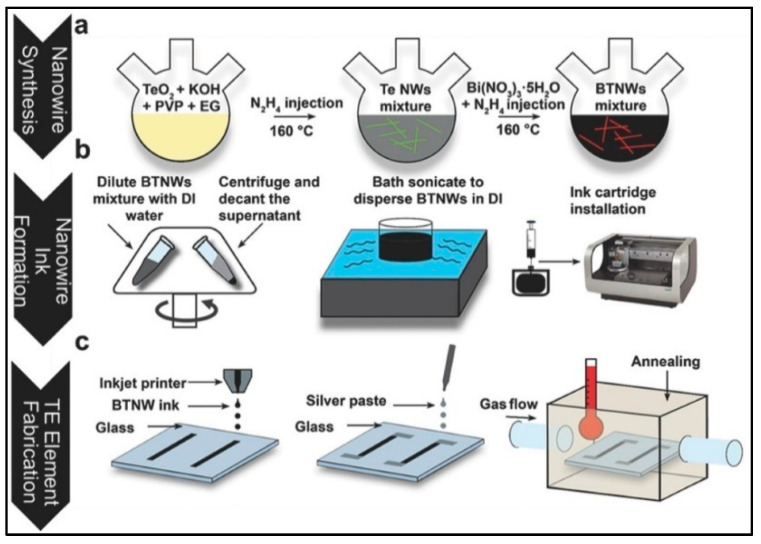
Schematic diagram of Bi_2_Te_3_ nanowire large-scale and low-cost synthesis based on inkjet printing: (**a**) Chemical batch processing, (**b**) Nanowire ink formation, and (**c**) Inkjet printing of the Bi_2_Te_3_ nanowires ink and post-annealing process. Reproduced with permission from [20]. Copyright Wiley Online Library, 2017.

The inkjet printing process is well-known as the method of printing images or texts onto a paper or porous surface. It is a solution-based, additive, non-contact, and mask-less deposition process in which high speed and resolution are used to pattern the materials [75]. In the past few decades, it is used as a free-form fabrication method for constructing 3D parts and is being explored as a means of printing flexible and wearable optical and electronic devices, especially when organic components are involved [76]. It is a maskless and non-vacuum process with a low implementation price, making it easier to integrate a printed device on a smart package [77]. There are mainly two types of technologies: drop-on and continuous demand. Inkjet printing in thermoelectrics is primarily used to deposit organic [78] or hybrid organic–inorganic materials [79,80], but some researchers have also used this method for inorganic thermoelectric synthesis. Firstly, in this method, firstly, the nanostructures are formed using processes such as solution synthesis and chemical synthesis. Then, the nanoparticles are mixed in an aqueous solution such as distilled water to form jettable ink. Then, this jettable ink is loaded onto a cartridge and dispersed through the nozzles on a printable substrate. This technique was first used for TE devices to fabricate n-type Bi_2_Te_2.7_Se_0.3_ nanoparticles and p-type Sb_1.5_Bi_0.5_Te_3_ nanoparticles [81]. Inks of aqueous Bi_2_Te_2.7_Se_0.3_ and Sb_1.5_Bi_0.5_Te_3_ were printed on silver electrodes in 150 layers and sintered at 250 °C for 30 min. Bismuth telluride thermoelectric nanowires prepared by chemical batch processing and converted into a jettable ink printed on a glass substrate yielded a Seebeck coefficient of 140 µVK^−1^ [20]. Figure 7a–c depicts the chemical batch processing, nanowire ink formation, and inkjet printing of the Bi_2_Te_3_ nanowires ink and post-annealing process. Another work reported an up-to-date highest output power of 127 nW at ΔT = 32.5 in an inkjet-printed TEG with p-type bismuth antimony telluride (Bi_0.5_Sb_1.5_Te_3_) and n-type bismuth telluride (Bi_2_Te) [82]. The current output power and performance of TEG prototypes are very low. Furthermore, much more enhancement in the thermoelectric properties is required to successfully integrate inkjet printing for large-scale production.

#### 3.5.2. Screen Printing

**Figure 8:**
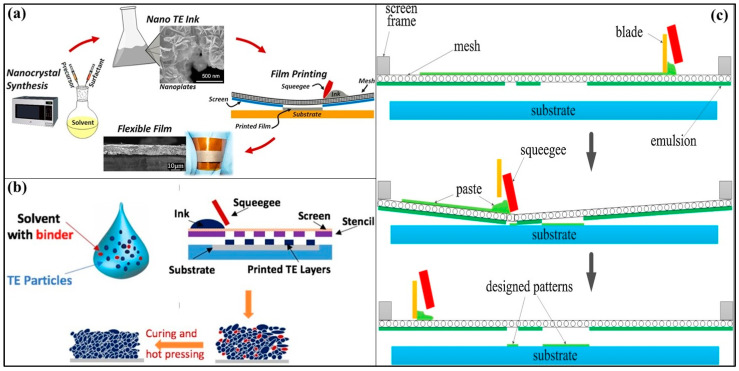

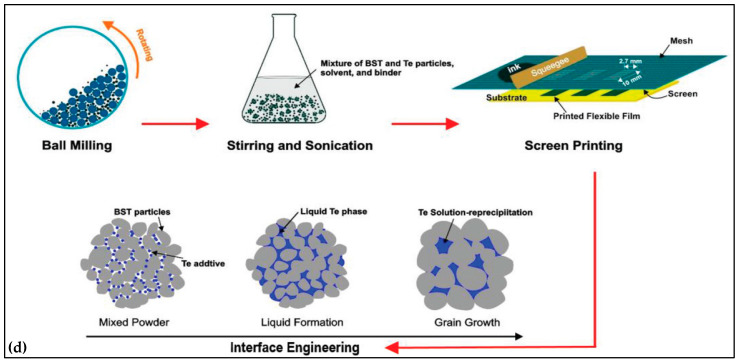
(**a**) Schematic illustration of the overall flexible n-type Bi_2_Te_2.8_Se_0.2_ TE films fabrication process from nanocrystal synthesis to sintered flexible films, including nano TE ink processing and screen printing on a flexible substrate. Reproduced with permission from [86]. Copyright Scientific reports, 2016, (**b**) Schematic illustration of the fabrication of flexible TE films with p-type Bi_0.5_Sb_1.5_Te_3_ and n-type Bi_2_Te_2.7_Se_0.3_. The printable ink, screen-printed TE layer, and hot-pressed layer (after screen printing) can also be seen. Reproduced with permission from [88]. Copyright Scientific reports, 2017, (**c**) Schematic illustration of pattern deposition for screen printed fabrication of flexible TE films with p-type Sb_2_Te_3_ powders and n-type Bi_1.__8_Te_3.2_ powders. Reproduced with permission from [87]. Copyright Elsevier, 2016, (**d**) Overall fabrication of BiSbTe flexible films (Bi_0.4_Sb_1.6_Te_1.3_) using a low-cost and scalable screen-printing process and post-printing nanosolder-assisted interface engineering. Reproduced with permission from [90]. Copyright Wiley Online Library, 2019.

Screen printing is a process that involves a screen mesh to print on the target substrates by passing ink through the screen mask mesh. This concept dates to the 1800s for printing canvases, fabrics, and textiles. Lately, with fine line printing development, screen printing is used in electronics for ultra-fine processing. Lee et al. [83] were the first to use this method to print a low-cost thermoelectric module with ZnSb as p-type and CoSb_3_ as an n-type material, and copper as the pad metal. The ZnSb films had an optimized carrier concentration of 7 × 10^18^/cm^3^ with the power density and output voltage of 0.17 mW/cm^2^ and 10 mV at ΔT = 50 K. A subsequent annealing process for the fabrication of Sb_2_Te_3_ thick films after screen printing improved the figure of merit to 0.32 at room temperature [84]. Screen printing bismuth telluride thick films by subsequent optimized annealing at 500 °C for 15 min resulted in a ZT value of 0.61 at room temperature [85]. In another work, n-type Bi_2_Te_2.8_Se_0.2_ nanocrystal ink was synthesized using the microwave-stimulated wet-chemical method and screen printed on a polyimide substrate (flexible) [86]. A maximum figure of merit of 0.43 was demonstrated with superior flexibility. Figure 8a shows the overall fabrication of flexible n-type Bi_2_Te_2.8_Se_0.2_ TE films from nano-ink processing to screen printing on the flexible polyimide substrate. Binder additives are essential for an effective and smooth printing process, but large quantities deteriorate the electrical transport properties of the TE layers. Two different binders, namely binder A (4,4′-isopropylidenediphenol-epichlorohydrin based epoxy (3 M)) and binder B (epichlorohydrin-polyglycol based epoxy (Dow Chemical)) in two distinct cases—cold isostatic pressing (CIP) and no CIP—were compared for their effect on the resistivity of the n-type BiTe and p-type SbTe thermoelectric pastes [87]. Figure 8c shows the illustration of pattern deposition for screen-printed fabrication of flexible TE films with p-type Sb_2_Te_3_ powders and n-type Bi_1.__8_Te_3.2_ powders. It was found that the optimum combination of materials was that SbTe with binder A, BiTe with binder B, along with SbTe electrodes, and the assembly prone to CIP. Another binder additive methylcellulose, used in screen printing a flexible TE film with p-type as Bi_0.5_Sb_1.5_Te_3_ and n-type Bi_2_Te_2.7_Se_0.3_, offered sufficient viscosity for printing at a minimal concentration (0.45–0.60 wt %) [88]. Figure 8b depicts the schematic illustration of the fabrication of flexible TE films with p-type Bi_0.5_Sb_1.5_Te_3_ and n-type Bi_2_Te_2.7_Se_0.3_. The methylcellulose binder burns off during the hot pressing and sintering, negating its effect on the features of the TE films and resulted in a maximum ZT of 0.65 and 0.81 for p-type and n-type, respectively, at room temperatures. Post annealing the screen-printed TE films in a forming gas ambient (4% H_2_ + 96% Ar) increased the ZT to two-folds (ZT = 0.90) when compared to the screen-printed films without annealing [89]. The performance of TE printed devices suffers from poor interfacial connectivity between the nanoparticles, leading to low carrier mobility. This shortcoming is tackled by a Te-based nanosolder approach to bridge the interfaces in BiSbTe NP during the sintering process after the printing [90]. This work demonstrated a maximum figure of merit of ≈1 for the screen-printed BiSbTe flexible films, which is up to date the maximum performance reached by any printing technique. The overall fabrication of BiSbTe flexible films (Bi_0.4_Sb_1.6_Te_1.3_) using a low-cost and scalable screen-printing process and post-printing nanosolder-assisted interface engineering is shown in Figure 8d. A novel single-step crystallization process done with a paste containing an excess of tellurium for screen printing resulted in an enhanced ZT (p-type—0.93 and n-type—0.64) in flexible TEGs consisting of 200 couples indicating superior reproducibility and reliability feasible for batch production of high-output TEGs [21]. Screen printing is a very simple and promising fabrication technique for the mass production of flexible thermoelectric generators with superior output powers compared to other printing techniques. Developing appropriate binders and post-printing techniques would further aid in boosting the throughput of the screen printing, improving the scalability of the TEG devices for various applications.

#### 3.5.3. Dispenser Printing

**Figure 9:**
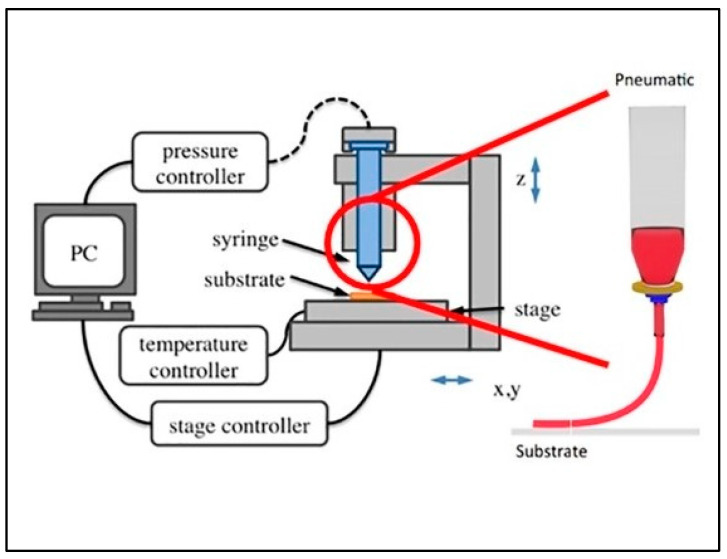
Schematic illustration of dispenser printer. Reproduced with permission from [75]. Copyright Wiley Online Library, 2016.

The use of a wide range of electrically functional inks for printing and ease in changing the design without considering the requirements for screens or masks makes dispenser printing a rapidly growing additive manufacturing process. More viscous materials can be used in this method compared to other techniques offering superior functionality of the printed layers. It is a drop-on-demand technology indicating dispensing of ink in the areas required. This allows optimized use of the materials reducing the environmental impact and printing of complex geometries. Depending on the nozzle used, the target materials are dispersed in a suitable binder to make them an appropriate viscosity for printing. This dispenser technique was first used to print TE thick films made of p-type Sb_2_Te_3_-epoxy and n-type Bi_2_Te_3_-epoxy with the figure of merit ZT of 0.41 and 0.16, respectively [22]. The output power for a 50-couple prototype device of size 5 mm × 640 µm × 90 µm was 10.5 µW for ΔT = 20 K, and the total areal power density of the device was 75 µWcm^−2^ [91]. Figure 9 depicts the schematic illustration of the fabrication of flexible TE films with p-type Sb_2_Te_3_-epoxy composite and n-type Bi_2_Te_3_-epoxy composite using planar dispenser printing. A 62 single-leg mechanical alloyed n-type Bi_2_Te_3_TEG prototype of dimensions 5 mm × 700 µm × 120 µm dispenser printed on a polyimide substrate produced an output power of 25 µW for ΔT = 20 K and ZT of 0.31 at 350 °C [92]. This same technique (dispenser printing + mechanical alloying) was extended to fabricate a p-type Bi_0.5_Sb_1.5_Te_3_ prototype of size 5 mm × 700 µm × 120 µm with an output power of 20.5 µW for ΔT = 20 K and ZT of 0.2 at 250 °C [93]. The output power was further increased to 130 µW at ΔT = 70 K in a ten couple TEG device with dispenser printing the mechanical alloyed p-type Bi_0.5_Sb_1.5_Te_3_ (with 8 wt % extra Te-epoxy composite) and n-type Bi-epoxy composite [94]. A non-contact dispenser printing method with selective laser melting (SLM) was used to fabricate n-type Bi_2_Te_2.7_Se_0.3_ single-phase thin layers that demonstrated a Seebeck coefficient of −152 µVK^−1^ [95]. These results show a promising future for dispenser printing to design scalable, low-cost TEGs for various applications.

#### 3.5.4. Aerosol Printing

**Figure 10:**
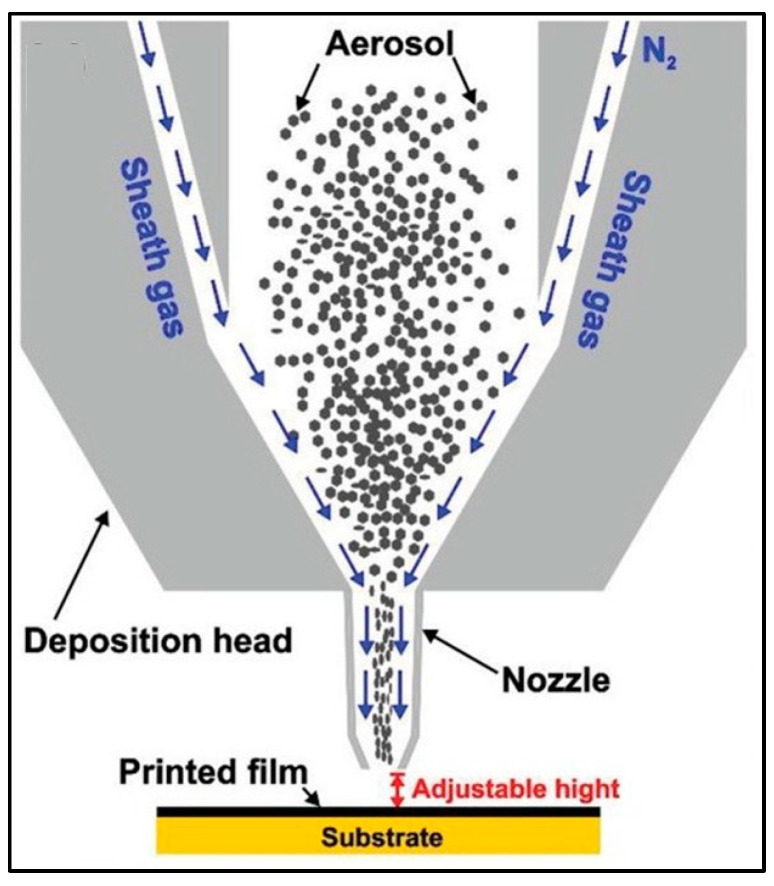
Schematic illustration of aerosol printing. Reproduced with permission from [97]. Copyright Wiley Online Library, 2019.

Aerosol printing/spray printing is another famous method for printable thermoelectrics. In this method, particles of size starting from 20 nm are aerosolized from ink through an atomizer. This method has the advantage of the possibility to print on non-smooth and non-flat substrates successfully. Ou et al. fabricated a TEG made from Sb_2_Te_3_ nanoflakes and carbon nanotubes with a power factor of ≈41 μWm^−1^ K^−2^ using a modified aerosol-jet printing method [96]. Dun et al. used this 3D conformal aerosol printing of solution-processed p-type Sb_2_Te_3_ to print flexible TEG with a high power factor of 2.2 mWm^−1^ K^−2^ at 500 K [97]. Figure 10 illustrates the 3D aerosol jet printing of p-type Sb_2_Te_3_. Another work used this method to fabricate highly scalable Bi_2_Te_2.7_Se_0.3_ with a power factor of 730 µWm^−1^ K^−2^ [98]. The aerosol printing method has also been used to fabricate organic materials and hybrid organic–inorganic TEGs [99,100].

#### 3.5.5. Photonic Sintering

**Figure 11:**
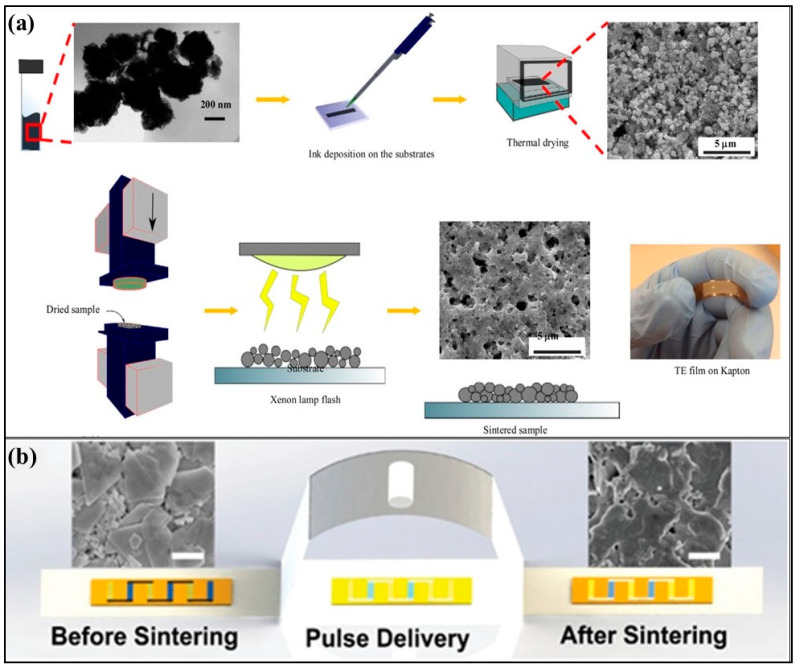
(**a**) Schematic illustration showing the Bismuth Telluride Thermoelectric film preparation by photonic sintering. Reproduced with permission from [104]. Copyright 2018, Advanced Engineering Materials. (**b**) Illustrating the effect of intense pulsed light sintering on Bi_2_Te_2.7_Se_0.3_ nanoplates with before sintering and after sintering SEM images. Reproduced with permission from [98]. Copyright Wiley Online Library, 2019.

The concept of using xenon flashlight was developed for application in printed electronics to quickly sinter copper nano-ink at room temperatures and ambient conditions on a low-temperature polymer substrate [101]. This method was also used to print silver nanoparticles on temperature-sensitive substrates to prepare highly conductive structures on foils [102]. Photonic sintering is advantageous to conventional sintering methods in higher conductivities, shorter processing times, better adhesion, and scalabilities [103]. Based on this concept, intense or concentrated UV rays from a Xenon lamp were used to sinter bismuth telluride thermoelectric films to achieve a conductivity of 3200 Sm^−1^ and a power factor of 30 µWm^−1^ K^−2^ [104]. Figure 11a shows the photonic sintering schematic illustration and scanning electron microscopy (SEM) images of the sample powder both after the thermal drying and after the photonic sintering. The photograph of the flexible TE film on the Kapton substrate can also be seen. This work is the first time photonic sintering was used for thermoelectric synthesis, and it sinters the TE nanoparticles within milliseconds under ambient conditions. In this process, firstly, the nanoparticle ink is created and then dispersed on a substrate, which is followed by thermal drying. Then, these dried samples were cold pressed and exposed to a xenon lamp flash for sintering. The photonic sintering of solution-processed Bi_2_Te_2.7_Se_0.3_ changed from non-conducting to a highly conductive state (2.7 × 10^4^ S-m^−1^) within seconds [98].

Figure 11b illustrates the effect of intense pulsed light sintering on Bi_2_Te_2.7_Se_0.3_ nanoplates before sintering and after sintering SEM images. Thus, photonic sintering is a very competent method for devising flexible thermoelectric devices as energy harvesters for portable and wearable electronics.

### 3.6. Other Methods

A facile, low-temperature, and green gram-scale route was proposed to synthesize ultrathin Bi_2_S_3_ necklace nanowires of thickness < 2 nm with colloidally stable and robust excitonic features [105]. Yang et al. [106] used the molten slat method to synthesize highly crystalline Bi_2_S_3_ nanowires of lengths ≈ 20 µm with better power factors. Another process for large-scale synthesis of Ternary BiSbTe films using DC magnetron sputtering and annealing has been proposed [107]. This work observed an increased power factor due to ascending and descending in Seebeck coefficient and electrical resistivity, respectively, with an increase in annealing temperature. Thin films of Cu_3_BiS_3_ were prepared by co-evaporation. The Hall effect, Seebeck effect, and surface photovoltage measurements show that Cu_3_BiS_3_ is a p-type semiconductor with Hall mobility, free carrier concentration, and thermo-electric power of 4 cm^2^/Vs, 2 × 10^16^ cm^−3^, and 0.73 mV/K, respectively [108]. Similar to these works, several novel methods are exploring this domain to produce highly efficient nanostructured TE on a large scale.

## 4. Limitations, Future Scope, and Conclusions

The wide application of thermoelectric generators relies primarily on two parameters: (1) materials that are low toxic and earth abundant, and (2) synthesis techniques that are simple, easy, low cost, quick, scalable, and reliable. The methods must enhance the thermoelectric properties of the materials and be replicable or reproducible. In this report, we highlight some notable works that have investigated large-scale synthesis techniques along with the benefits and drawbacks of each method. The effect these methods have on the thermoelectric properties of the materials is also reviewed. There is room for further improvement in better optimization and increased performance, even with the astonishing progress over the last few decades.

Breakthrough advancement in nanomaterials facilitating the fabrication of highly efficient TEG/devices will be achieved by better understanding phenomena and mechanisms at the atomic level and better control of material parameters. Simultaneously, the growing industrial innovations will necessarily support and provide cost-effective technologies capable of transforming this understanding and control into optimized products. The synthesis and fabrication methods mentioned above would assist in this direction and provide TE nanomaterials with specifically modulated parameters to develop cost-effective, high-yield, and high-throughput TE devices. However, there are certain limitations these methods offer that must be overcome. The material parameter control achieved at the nanoparticle level is far from scaling it to the macroscopic level. Currently available consolidation processes have led to crystal domain growth, atomic redistribution, stoichiometric changes, phase or alloying segregation, and interface redefinition. A significant advancement in the surface chemistry engineering of nanoparticles is necessary to overcome these issues for better charge transport properties. Moreover, TE applications at high temperatures require extraordinary stabilization processes that avoid any unstable nanostructured assemblies.

The critical limitation of nanoparticle-based processes is difficulty in scaling and reproducing the materials with the same and exact features. To address this issue, significant improvements in the simple and straightforward engineering and design of the TE materials are required. These improvements must also accommodate the cost and volume of the highly functional nanomaterials. Another barrier in engineering high yield TE nanocomposites is the inferior understanding available on the transport features of the complex systems. Information on the precise effect of phase distributions, interfaces, quantum confinement, and crystal domain size on the performance is necessary. Better computational modeling, along with a large number of experimental characterizations of system models with tuned parameters, can assist in this aspect. The performance of TE materials is essentially measured by the figure of merit value the product material achieves. This belief in some way has restricted the growth of novel materials and processing techniques.

In printed TEGs, layer adhesion and crack formation are common difficulties that restrict the production of dense layers with adequate thickness. The nascent 3D printing technology offers some relief in overcoming these difficulties. However, it remains to be seen whether these technologies would suffice the throughput requirements of the industries. Another thing is that the materials that are to be designed should consider their most plausible final application. Even with optimum cost and efficiencies, the thermoelectrics cannot compete on a large scale with the existing steam engines or compressor-based refrigerators. The thing that makes thermoelectrics unique is the very accurate temperature control they provide, the capability to harvest small temperature gradients, their portability, their wide scalability, and their effectiveness at a very small scale. So, to summarize, the future of TE strongly relies on our capability to develop novel materials with optimized performance. The various methods discussed above can considerably contribute to this goal, while at the same time, we need to improve the better understanding of the transport properties of the nanomaterials and control of the specified parameters.

## Figures and Tables

**Table 2:** Summary of recent progress in prominent large-scale fabrication techniques.

Technology	Materials	Processing Cost	Time	Scalability (Max Weight Per Batch)	Ztmax (Material-Ref)	Strengths	Drawbacks
Inkjet printing	Organic, hybrid organic–inorganic	Low	Medium	High	0.26 (Bi_2_Te_3_ [20])	High quality, fine, and smooth printing. Power source in wearable and portable electronics	Nozzle clogging, nozzle plate flooding, and erratic droplet ejection
Screen printing	Organic, hybrid organic–inorganic	Low	Fast	High	≈1(Bi_0.5_Sb_1.5_Te_3_ [21])	Printable on diverse substrates, durable and high quality	Relatively complex and less eco-friendly
Dispenser Printing	Organic, hybrid organic–inorganic	Low	Medium	Medium	0.41 (Sb_2_Te_3_-epoxy [22])	Higher contact resistance, simple, easy, and do not have post-processing requirements	Slow dispensing and difficulty in reproducibility
Photonic sintering	Inorganic (Bismuth-based)	Low	Fast	High	-	Higher conductivities, shorter processing times, better adhesion, flexibility	The intense light pulses lead to increased energy consumption

## Data Availability

No data available.
